# Inhibition of Netosis with PAD Inhibitor Attenuates Endotoxin Shock Induced Systemic Inflammation

**DOI:** 10.3390/ijms232113264

**Published:** 2022-10-31

**Authors:** Huanling Yao, Guojie Cao, Zheng Liu, Yue Zhao, Zhanchao Yan, Senzhen Wang, Yuehua Wang, Zhengwei Guo, Yanming Wang

**Affiliations:** Laboratory of Epigenetics and Translational Medicine, School of Life Sciences, Henan University, Kaifeng 475004, China

**Keywords:** PAD inhibitor, endotoxin shock, netosis, systemic inflammation, LPS, RNA-seq

## Abstract

Neutrophils play a pivotal role in innate immunity by releasing neutrophils extracellular traps (NETs). Excessive NETs are detrimental to the local tissue and further exacerbate inflammation. Protein arginine deiminases (PAD) mediate histone citrullination and NET formation that, in turn, exacerbate endotoxin shock damages. In this study, we further investigated the molecular mechanism underlying PAD and NETs in endotoxic stress in mice. The control group mice were injected with solvent, the LPS endotoxic shock group mice were intraperitoneally injected with LPS at 35 mg/kg only, while the LPS and PAD inhibitor YW3-56 treatment group mice were injected with YW3-56 at 10 mg/kg prior to the LPS injection. YW3-56 significantly prolonged the survival time of the LPS-treated mice. NETs, cfDNA, and inflammatory factors were detected by ELISA in serum, paitoneal cavity, and lung at 24 h after LPS administration. Lung injuries were detected by immunostaining, and lung tissue transcriptomes were analyzed by RNA-seq at 24 h after LPS administration. We found that YW3-56 altered neutrophil tissue homeostasis, inhibited NET formation, and significantly decreased cytokines (IL-6, TNFα and IL-1β) levels, cytokines gene expression, and lung tissue injury. In summary, NET formation inhibition offers a new avenue to manage inflammatory damages under endotoxic stress.

## 1. Introduction

Neutrophils are the most abundant white cells in circulating blood and form the first line of defense against invading pathogens [1]. Neutrophils express a diverse range of toll like receptors (TLRs) that recognize and respond to pathogen-associated molecular patterns (PAMPs) or damage-associated molecular patterns (DAMPs) [2]. Lipopolysaccharides (LPS) are primary membrane components of gram-negative bacteria, which bind to TLR4 to activate various immune responses [3].

Neutrophils play a pivotal role in killing and digesting bacteria and fungi at the infection site to prevent severe tissue damage and septic complications and help tissue repair. In addition to phagocytosis-mediated intracellular killing, neutrophils also mediate extracellular killing of bacteria via a chromatin-based neutrophil extracellular trap (NET) structure [4]. NETs are composed of genomic DNA and histones in addition to various antimicrobial factors, such as neutrophil elastase and myeloid peroxidase [5]. Although the antimicrobial functions of NETs are critical for host defense and innate immunity, NETs released into the tissues nonetheless damage surrounding health tissues and exacerbate local and systemic inflammation. As such, to control NET formation and clearance, homeostasis is important for individual health.

Peptidylarginine deiminases (PADs) are a family of peptidylarginine-modifying enzymes that convert arginine residues to citrulline [6]. The PAD protein family in humans and mice contains five members, including PAD1–4 and PAD6 [6,7]. PAD1 mainly regulates the cornification of epidermal cells [8]. PAD2 is found in many tissues, including muscles, brain, and secretory glands, and can regulate myelin basic protein, vimentin, and actin [6,7,9].

Of the five PAD family members, PAD4 is highly expressed in neutrophils and other myeloid lineage cells [10]. Histone hypercitrullination catalyzed by PAD4 plays a pivotal role in the formation of NETs, thereby regulating innate immunity to contribute to the bactericidal process [11]. On the other hand, excessive PAD4 activity also contributes to disease etiology involving NETs, such as malignant tumors and rheumatoid arthritis [12,13]. A role of NETs in endotoxic shock and sepsis is well established [14,15,16,17]. However, the mechanism by which PAD4 and its family members activate in severe endotoxin stress and systemic inflammation remains largely unknown. Luo et al. designed an irreversible substrate analogue covalent inhibitor Cl-amidine based on a chloroacetamidine warhead, which has an enzymatic IC50 of ~5.9 μM against human PAD4 [18]. To improve the cell membrane permeability and enzymatic activity of this inhibitor, our group has synthesized a series of compounds, including YW3-56, with higher membrance permeability and enzymatic inhibiton acitivity against human PAD4 with an IC50 about 1.19 μM [19].

Here, using a PAD enzyme inhibitor YW3-56 as a tool, we investigated the molecular mechanism underlying PAD and NETs in endotoxic stress in mice.

## 2. Results

### 2.1. Chloroacetamide Based PAD Inhibitor YW3-56 Inhibits Histone Citrullination and NET Formation

**Figure 1:**
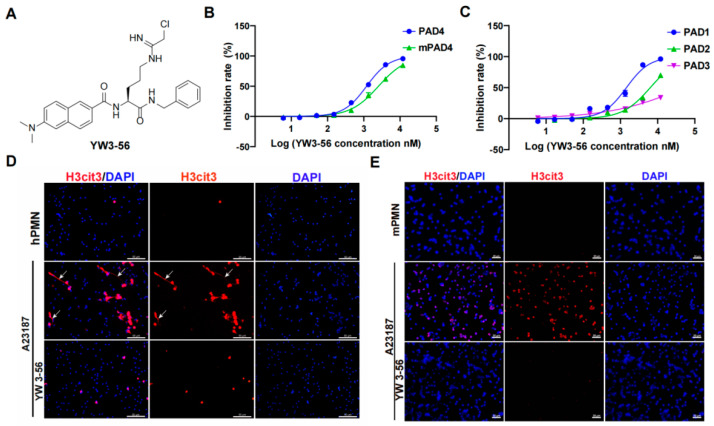
PAD inhibitor YW3-56 inhibits histone citrullination and NET formation. (**A**) YW3-56 is designed based on a structural similarity with the substrate of PAD family peptidylarginine. (**B**) YW3-56 inhibits mouse PAD4 and human PAD4 activity with IC50 of 2.54 × 10^3^ nM and 1.19 × 10^3^ nM. (**C**) YW3-56 inhibits PAD1, 2, and 3 with IC50 of 1.45 × 10^3^ nM, 6.34 × 10^3^ nM, and 53.43 × 10^3^ nM. (**D**) Upon calcium ionophore treatment, human peripheral blood neutrophils increased histone citrullination (red) and NET formation (white arrow) detected by immunostaining with a histone H3Cit (Cit2, 8, 17) antibody. DNA dye DAPI staining in blue. NET formation and histone citrullination were decreased upon YW3-56 treatment. (**E**) Mouse bone marrow neutrophils showed a dramatic increase of histone citrullination after calcium ionophore treatment. YW3-56 treatment blocked the increase of histone citrullination. Scale bars represent 50 μm. hPMN, human polymorphonuclear cell; mPMN, mouse polymorphonuclear cell; PAD, peptidylarginine deiminase. NET, neutrophil extracellular trap.

YW3-56 is designed based on a structural similarity with the substrate of PAD family peptidylarginine (Figure 1A) [19]. YW3-56 inhibits mouse PAD4 and human PAD4 with IC50 of 2.54 × 10^3^ nM and 1.19 × 10^3^ nM, respectively (Figure 1B), indicating it is an efficient PAD4 inhibitor. In addition to PAD4, the PAD family has three other active enzymes, including PAD1, 2, and 3. YW3-56 inhibited PAD1, 2, and 3 with IC50 of 1.45 × 10^3^ nM, 6.34 × 10^3^ nM, and 53.43 × 10^3^ nM, respectively (Figure 1C), indicating that YW3-56 inhibits all members of the PAD family at different degrees of efficacy.

We next tested the efficacy of YW3-56 to inhibit histone citrullination and netosis in human and mouse neutrophils. Human peripheral blood neutrophils from healthy donors showed very low levels of histone citrullination and NET formation (Figure 1D, top panels, Figures S1A and S2A, top panels). Upon treatment of calcium ionophore to increase the intracellular levels of calcium, a significant increase of histone citrullination was detected in human neutrophils (Figure 1D, middle panels, Figure S1A). It is notable that human neutrophils released extensively decondensed extracellular chromatin (Figure 1D, middle panels, denoted by arrows). In contrast, treatment with YW3-56 before calcium ionophore effectively inhibited histone citrullination, as well as netosis (Figure 1D, lower panels, Figure S1A). Furthermore, histone citrullination in mouse bone marrow neutrophils induced by calcium ionophore was also significantly decreased after YW3-56 treatment (Figure 1E, lower panels, Figure S1B). Although mouse bone marrow neutrophils undergo netosis, the degree of chromatin release and decondensation was much lower (Figure 1E, middle panels).

Upon treatment of LPS, an increase of histone citrullination was also detected in human neutrophils (Figure S2A, middle panels). In contrast, treatment with YW3-56 prior to LPS effectively inhibited histone citrullination as well as netosis (Figure S2A, lower panels). Furthermore, histone citrullination in mouse bone marrow neutrophils induced by LPS was also significantly decreased after YW3-56 treatment (Figure S2B, lower panels).

### 2.2. YW3-56 Decreased Endotoxin LPS-Induced Mouse Death and Serum NETs, cfDNA, TNFα, IL-6, IL-1β Levels

**Figure 2:**
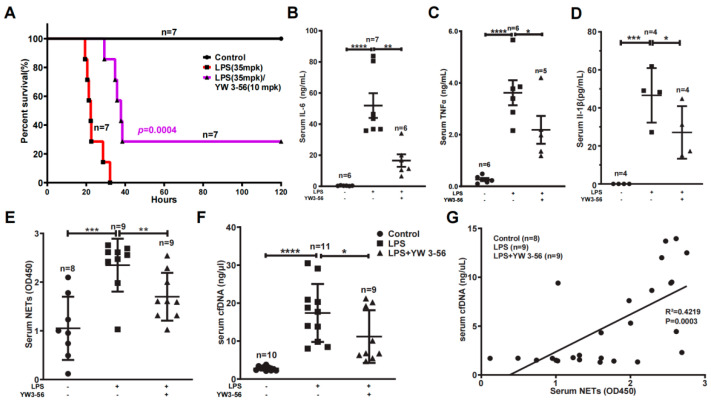
PAD inhibitor YW3-56 decreases endotoxic mouse death and serum NETs, cfDNA, TNFα, IL-6 and IL-1β levels. (**A**) In lethal LPS (35 mpk) induced endotoxic stress model, treatment with YW3-56 (10 mpk) decreased LPS mediated mouse death (*n* = 7 in each group). (**B**–**F**) Serum IL-6 levels (*n* = 6, 7, 6 in each group) (**B**), TNFα levels (*n* = 6, 6, 5 in each group) (**C**), IL-1β levels (*n* = 4 in each group) (**D**), NETs levels (*n* = 8, 9, 9 in each group) (**E**), and cfDNA levels (*n* = 10, 11, 9 in each group) (**F**) were much increased after LPS treatment, while YW3-56 treatment significantly decreased serum IL-6, TNFα, IL-1β, NETs and cfDNA levels. (**G**) Serum cfDNA levels had a strong correlation with the serum NET levels. The Person’s correlation analysis was used in survival analysis. All data in figures were presented as mean ± SEM. * *p* < 0.05; ** *p* < 0.01; *** *p* < 0.001; **** *p* < 0.0001. IL, interleukin; TNF, tumor necrosis factor; NET, neutrophil extracellular trap; cfDNA, cell free DNA; LPS, lipopolysaccharides.

We found that LPS at 35 mg/kg dosage essentially induced mouse death at a rate of one hundred percent, with a median survival time of 22 h (Figure 2A). In contrast, administration of one dose of YW3-56 prior to LPS treatment decreased the lethality to 70%, and the median survival time was increased to 38 h (Figure 2A). We next analyzed the effect of YW3-56 on inflammation factors in the serum. IL-6 levels were increased to above 50 ng/mL in serum after endotoxic induction (Figure 2B). In contrast, IL-6 levels were decreased to below 20 ng/mL in serum with YW3-56 treatment before LPS administration (Figure 2B). Likewise, TNFα levels were dramatically increased to an average of 3.5 ng/mL in endotoxic stress response after LPS administration (Figure 2C), while YW3-56 pretreatment decreased serum TNFα to ~2 ng/mL (Figure 2C). IL-1β levels were increased to an average of 47 pg/mL in serum after endotoxic induction with LPS, and YW3-56 pretreatment before LPS administration decreased serum IL-1β to ~27 pg/mL (Figure 2D).

After netosis, neutrophils can release chromatin NETs into the circulating blood. In mice with LPS-induced endotoxin stress, serum NETs levels were greatly increased, while YW3-56 treatment prior to LPS administration decreased NETs levels (Figure 2E). Similarly, serum cell-free DNA (cfDNA) levels significantly increased in mice with LPS-mediated endotoxic stress (Figure 2F). The increase of cfDNA was suppressed in mice with YW3-56 pretreatment prior to LPS administration (Figure 2F). Moreover, serum NETs and cfDNA levels are correlated (Figure 2G), suggesting that netosis contributes at least partially to the cfDNA in the circulating blood.

### 2.3. YW3-56 Inhibited Netosis, Decreased TNFα, IL-6 and cfDNA Levels and Increased the Amounts of Neutrophils in Peritoneal Cavity

**Figure 3:**
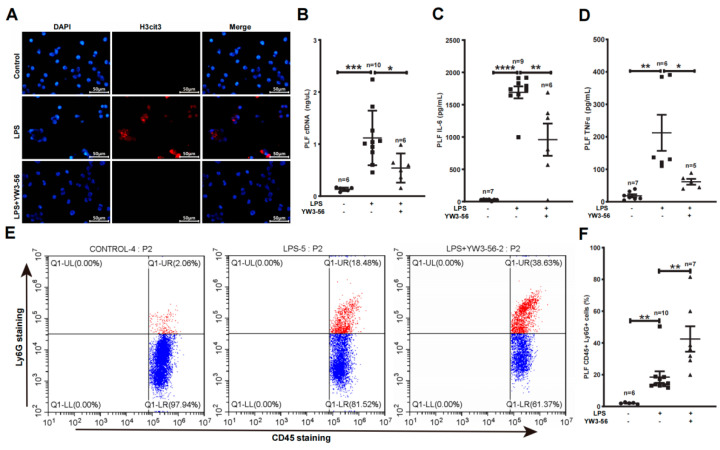
PAD inhibitor YW3-56 decreased netosis, cfDNA, IL-6 and TNFα levels and led to a high amount of neutrophil accumulation in peritoneal cavity. (**A**) After LPS treatment, neutrophils obtained from peritoneal lavage fluid (PLF) showed an increased histone citrullination (red) and netosis as detected by immunostaining with a histone H3Cit (Cit2, 8, 17) antibody and DNA dye DAPI staining (blue). NET formation and histone citrullination were decreased upon YW3-56 treatment. (**B**–**D**) PLF cfDNA (*n* = 6, 10, 6 in each group) (**B**), IL-6 (*n* = 7, 9, 6 in each group) (**C**) and TNFα (*n* = 7, 6, 5 in each group) (**D**) levels were much increased after LPS treatment, while YW3-56 treatment significantly decreased cfDNA, IL-6 and TNFα levels. (**E**,**F**) Percentage of neutrophils (*n* = 6, 10, 7 in each group) (CD45+ and Ly6g+) was significantly increased after LPS treatment, while YW3-56 treatment further neutrophil percentage. Scale bars represent 50 μm. All data in figures were presented as mean ± SEM. * *p* < 0.05; ** *p* < 0.01; *** *p* < 0.001; **** *p* < 0.0001. IL, interleukin; TNF, tumor necrosis factor; NET, neutrophil extracellular trap; cfDNA, cell free DNA; LPS, lipopolysaccharides; PLF, peritoneal lavage fluid.

We next analyzed NET formation and inflammatory factors within the peritoneal cavity—the site of LPS administration. We detected an increase of histone citrullination and netosis of neutrophils obtained from peritoneal lavage fluid (PLF) after LPS treatment in immunostaining experiments (Figure 3A, middle panels). In contrast, YW3-56 treatment prior to LPS administration decreased histone citrullination and netosis of PLF neutrophils (Figure 3A, bottom panels). Inflammatory factors, IL-6 and TNFα levels in PLF were much increased after LPS endotoxic stress (Figure 3C,D). In contrast, YW3-56 treatment prior to LPS administration significantly decreased IL-6 and TNFα levels in PLF (Figure 3C,D). Since YW3-56 inhibited netosis, we further analyzed the amount of neutrophil in PLF using flow cytometry. Among CD45+ leukocyte in PLF, the percentages of Ly6g+ neutrophils were increased from ~2% in normal mice to ~18% after LPS endotoxin shock (Figure 3E,F). Remarkably, YW3-56 treatment in mice under LPS endotoxin shock further increased Ly6g+ neutrophils to ~42%. Although high amounts of neutrophils are present, PLF cfDNA levels were decreased in the YW3-56 and LPS double treatment group compared with the LPS endotoxin shock group of mice (Figure 3B). The above results support a notion that netosis inhibition increases the number of neutrophils in PLF while simultaneously decreasing the production of inflammation factors such as IL-6 and TNFα.

### 2.4. YW3-56 Inhibits Endotoxin Shock Induced Lung Inflammation

**Figure 4:**
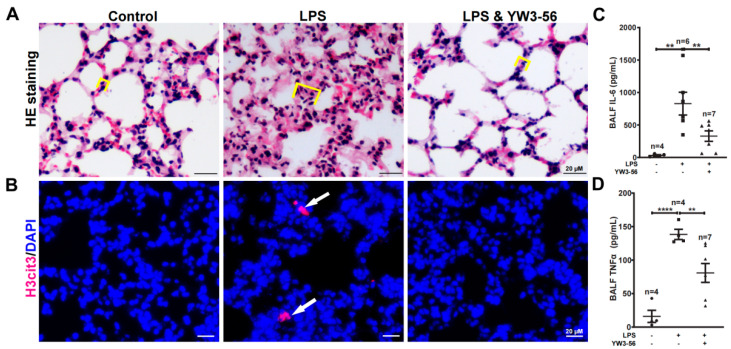
YW3-56 treatment decreased histone citrullination in lung sections, alveolar wall thickening, as well as IL-6 and TNFα levels in bronchoalveolar lavage fluid (BALF). (**A**) After LPS treatment, alveolar wall was much thickened analyzed with H&E staining. YW3-56 treatment largely reversed alveolar wall (yellow box line marked) thickening. (**B**) After LPS treatment, histone citrullination (red) was increased in lung sections detected by immunostaining with a histone H3Cit (Cit2, 8, 17) antibody and DNA dye DAPI staining (blue). Histone citrullination was decreased upon YW3-56 treatment. (**C**,**D**) BALF IL-6 (*n* = 4, 6, 7) and TNFα (*n* = 4, 4, 7) levels were much increased after LPS treatment, while YW3-56 treatment significantly decreased IL-6 and TNFα levels. Scale bars represent 20 μm. All data in figures were presented as mean ± SEM. ** *p* < 0.01; **** *p* < 0.0001. IL, interleukin; TNF, tumor necrosis factor; NET, neutrophil extracellular trap; cfDNA, cell free DNA; LPS, lipopolysaccharides; BALF, bronchoalveolar lavage fluid.

Systemic inflammation induced by septic shock often leads to lung injury thereby increasing lethality [20]. To analyze the effects of YW3-56 on lung inflammation, we performed pathology analyses of lung sections by H&E staining. The alveolar wall was thickened over two folds, and this increase induced by LPS was largely reversed by YW3-56 treatment (Figure 4A). Moreover, immunostaining of lung sections identified histone H3 citrullination positive nuclei in the LPS treatment group but rarely in lung sections of untreated or LPS and YW3-56 double treatment mice (Figure 4B). The levels of IL-6 and TNFα in bronchoalveolar lavage fluid (BALF) were increased after LPS endotoxin shock induction (Figure 4C,D). This increase of IL-6 and TNFα was much reduced in the LPS and YW3-56 dual-treatment group (Figure 4C,D). Taken together, the above results suggest that LPS treatment induced inflammation and lung tissue edema can be reversed by YW3-56 treatment.

### 2.5. YW3-56 Reversed the Expression of Inflammatory Genes Elevated in Lung Tissues by LPS

**Figure 5:**
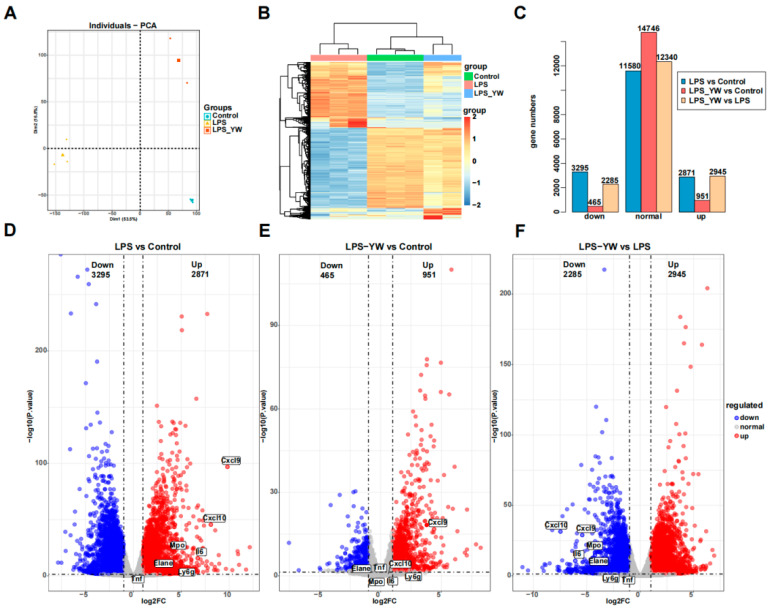
RNA-seq data analysis of lung tissues of mice from different treatment groups. (**A**) Principal component analysis found that there was no overlap between the three groups (Control group, *n* = 3; LPS group, *n* = 3; LPS/YW3-56 dual-treatment group, *n* = 2). (**B**) Hierarchical clustering of the 1000 most variable genes also demonstrated clear separation of three group of samples. (**C**,**D**) Differential expression analysis identified significant numbers of differential expressed genes in LPS relative to control samples (total 6166 genes with 2871 upregulated and 3295 downregulated). (**C**,**E**) Less numbers of differential expressed genes (1416 genes) in LPS and YW3-56 dual treatment relative to control samples were found, of which 951 genes were upregulated and 465 genes were downregulated. (**C**,**F**) 5230 of differential expressed genes in LPS and YW3-56 dual treatment relative to LPS treatment samples were found, of which 2924 genes were upregulated and 2285 genes were downregulated. PCA, principal component analysis; log2FC, log2 fold change; LPS, lipopolysaccharides treatment gruoup, LPS_YW, LPS and YW3-56 dual-treatment group.

To further analyze the effects of YW3-56 on lung inflammation, we performed transcriptomic analyses of lung tissue by RNA-seq. Initial exploratory data analysis by principal component analysis found that there was no overlap between the three groups (Control group, LPS group, LPS and YW3-56 dual-treatment groups), the internal data of each group was relatively reproducible (Figure 5A). Hierarchical clustering of the 1000 most variable genes also demonstrated clear separation of three groups samples (Figure 5B). The 1000 most variable genes showing expression changes among the three groups can be divided into four clusters (Figure 5B). The majority of genes are in clusters 1 and 2. Genes in cluster 1 showed activation by LPS but less activation or repression after YW3-56/LPS dual treatment, while genes in cluster 2 showed strong repression by LPS but weak repression by YW3-56/LPS dual treatment (Figure 5B). Gene expression patterns of the LPS and YW3-56 dual-treatment group are closely correlated with the control group than the LPS treatment group (Figure 5A,B).

Differential expression analysis identified significant numbers of differential expressed genes (DEG) in LPS relative to control samples, but much fewer numbers of differential expressed genes in LPS and YW3-56 dual treatment relative to control samples. 6166 genes expression were significantly altered in the lung tissues of mice with LPS-induced endotoxin shock compared to control groups (Figure 5C,D). Among them, 2871 genes were upregulated and 3295 genes were downregulated. However, there were only 1416 genes expression showing significant changes in the LPS and YW3-56 dual-treatment group when compared to control lungs (Figure 5C,E), of which, 951 genes were upregulated and 465 genes were downregulated. These results indicated that YW3-56 partially reversed the gene expression patterns induced by LPS, shifting the genes expression patterns in YW3-56/LPS treatment lungs tissues to those of normal lung tissue.

**Figure 6:**
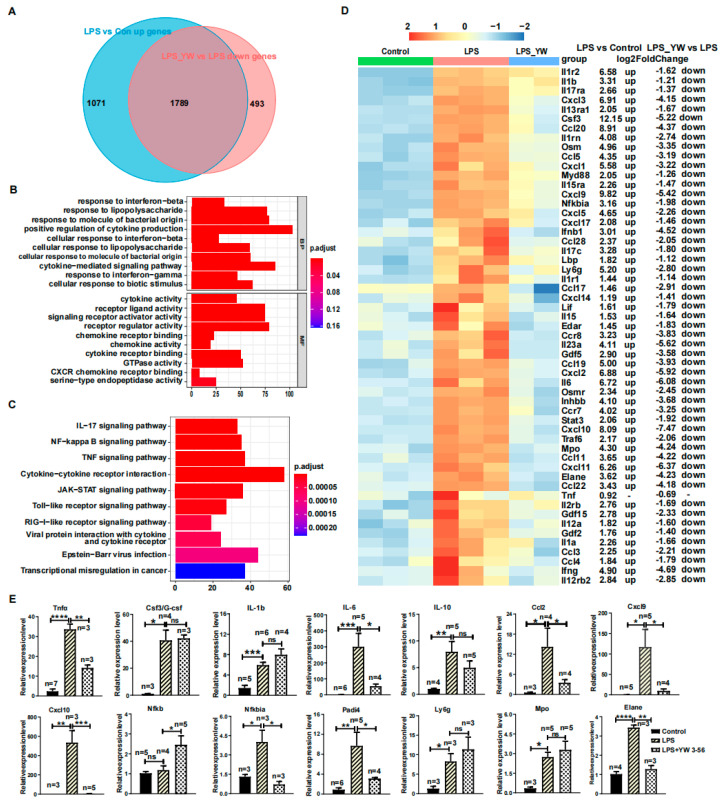
YW3-56 reversed LPS-induced upregulation of inflammation-related genes. (**A**) There were 1789 genes in the intersection of upregulated genes in LPS relative to control samples and downregulated genes of LPS and YW3-56 dual treatment relative to LPS treatment samples. (**B**) These 1789 genes were mainly enriched for biological processes such as positive regulation of cytokine production, cytokine-mediated signaling pathway, etc., and enriched for molecular functions including cytokine and cytokine receptor activity, chemokine and chemokine receptor activity, etc. (**C**) These 1789 genes were enriched in KEGG functional pathways including cytokine-cytokine receptor interaction, TNF signaling, JAK-STAT signaling, NF-kappa B signaling, IL-17 signaling etc. (**D**) The upregulated cytokines (*Il1a*, *Il1b*, *Il1rn*, *Il1f9*, *Il6*, *Il12a*, *Il15*, *Il17c*, *Il23a*, *Ifnb1*, *Ifng*, *Csf3*, *Lif*, *Osm*, *Gdf2*, *Gdf5*, *Gdf15*, *Inhbb*) and cytokines receptor (*Il1r1*, *Il1r2*, *Il2rb*, *Il12rb2*, *Il13ra1*, *Il15ra*, *Il17ra*, *Osmr*, *Edar*), chemokines (*Ccl3*, *Ccl4*, *Ccl5*, *Ccl11*, *Ccl17*, *Ccl19*, *Ccl20*, *Ccl22*, *Ccl28*, *Cxcl1*, *Cxcl2*, *Cxcl3*, *Cxcl5*, *Cxcl9*, *Cxcl10*, *Cxcl11*, *Cxcl14*, *Cxcl17*) and chemokines receptors (*Ccr7*, *Ccr8*), NF-kappa B, IL-17 and Toll-like receptor signaling pathway genes (*Nfkbia*, *Myd88*, *Lbp*, *Traf6*) were reversed by YW3-56. The upregulated neutrophil marker genes (*Elane*, *Mpo*, *Ly6g*) caused by LPS were also reversed by YW3-56. (**E**) Quantitative-PCR analysis confirmed that the upregulated gene expression by LPS, including *Il6*, *Ccl2*, *Cxcl9*, *Cxcl10*, *Nfkbia*, and *Elane.* was reversed by YW3-56 (*n* ≥ 3). All data in figures were presented as mean ± SEM. * *p* < 0.05; ** *p* < 0.01; *** *p* < 0.001; **** *p* < 0.0001.

To further identify the genes whose upregulated expression induced by LPS was reversed by YW3-56, a total of 1789 genes were obtained from the intersection of upregulated genes in LPS compared to control samples and downregulated genes of LPS/YW3-56 dual treatment compared to LPS groups (Figure 6A). Gene ontology enrichment analysis demonstrated that the primary biological processes in these 1789 genes involved positive regulation of cytokine production, cytokine-mediated signaling pathway, response to molecule of bacterial origin, response to LPS, and cellular response to biotic stimulus (Figure 6B). Molecular function included receptor regulator activity, signaling receptor activator activity, receptor ligand activity, cytokine activity and cytokine receptor binding, and chemokine activity and chemokine receptor binding (Figure 6B). These 1789 genes were also annotated with KEGG. Enriched functional annotations included cytokine-cytokine receptor interaction, TNF signaling pathway, JAK-STAT signaling pathway, NF-kappa B signaling pathway, IL-17 signaling pathway, and Toll−like receptor signaling pathway (Figure 6C). The upregulated cytokines (*Il1a*, *Il1b*, *Il1rn*, *Il1f9*, *Il6*, *Il12a*, *Il15*, *Il17c*, *Il23a*, *Ifnb1*, *Ifng*, *Csf3*, *Lif*, *Osm*, *Gdf2*, *Gdf5*, *Gdf15*, *Inhbb*) and cytokines receptor (*Il1r1*, *Il1r2*, *Il2rb*, *Il12rb2*, *Il13ra1*, *Il15ra*, *Il17ra*, *Osmr*, *Edar*), chemokines (*Ccl3*, *Ccl4*, *Ccl5*, *Ccl11*, *Ccl17*, *Ccl19*, *Ccl20*, *Ccl22*, *Ccl28*, *Cxcl1*, *Cxcl2*, *Cxcl3*, *Cxcl5*, *Cxcl9*, *Cxcl10*, *Cxcl11*, *Cxcl14*, *Cxcl17*) and chemokines receptors (*Ccr7*, *Ccr8)*, NF-kappa B, IL-17, and Toll-like receptor signaling pathway genes (*Nfkbia*, *Myd88*, *Lbp*, *Traf6*) were reversed by YW3-56 (Figure 6D). The upregulated neutrophils’ marker genes (*Elane*, *Mpo*) involved in NET formation and *Ly6g* induced by LPS were also reversed by YW3-56 (Figure 6D). Consistent with this notion, genes highly expressed after LPS treatment, including cytokines *Il.-6* and *Csf3*, chemokines *Cxcl9* and *Cxcl10*, and neutrophil marker genes *Elane*, *Mpo* and *Ly6g*, were decreased after YW3-58 treatment to levels that are not significantly different from those in normal lungs (Figure 5D,E). Quantitative-PCR analysis confirmed that the upregulated genes by LPS (*Il6*, *Ccl2*, *Cxcl9*, *Cxcl10*, *Nfkbia*, and *Elane*) were reversed by YW3-56 (Figure 6E).

## 3. Discussion

Here, we found that inhibition of NET formation decreased inflammation in the peritoneal cavity at the LPS injection site, as well as in the blood and lung at the site distant from LPS injection. NETs inhibition leads to a higher number of neutrophils accumulated at the primary inflammatory sites and peripheral blood, while at the same time, leads to decreased TNFα, IL-6, and IL-1β production. Using RNA-seq and bioinformatics analyses, we analyzed the gene expression patterns in lungs of normal, LPS stimulated, and LPS-PAD4 inhibitor dual-treated mice. Gene expression analyses indicate that LPS-induced inflammatory gene expression profiles largely reversed after PAD inhibitor treatment.

Netosis increased inflammatory response by promoting the production of IL-6, TNFα, and IL-1β in serum, peritoneal cavity, and lungs (Figure 2B–D, Figure 3C,D and Figure 4C,D). In contrast, netosis inhibition by a pan PAD inhibitor YW3-56 reversed the inflammatory factor production and alleviated the complications caused by endotoxic shock caused by LPS (Figure 2B–D, Figure 3C,D and Figure 4C,D).

Not only in the LPS administration site—the peritoneal cavity—did chemotaxis lead to an increased number of infiltrating neutrophils (Figure 3F), but also in peripheral blood, the proportion of neutrophils increased (Figure S3A). It has been reported that the number of peripheral lymphocytes is significantly reduced because of apoptosis in sepsis models and patients [21]. Our results show that the apoptosis of neutrophils decreased after LPS treatment, and YW3-56 treatment did not affect the apoptosis (Figure S3B,C). HMGB1 (high mobility group protein B1), which is the first molecule identified as DAMPs, could activate innate immune cells to propagate pro-inflammatory signaling cascades and induce recruitment of neutrophils to the site of tissue injury [22,23,24]. HMGB1 levels in serum increased after LPS treatment (Figure S4), which is consistent with that of septic patients [25,26]. This indicates that LPS treatment decreases neutrophils apoptosis but increases neutrophils infiltration; more neutrophils undergo netosis, producing NETs and aggravating the systemic inflammatory response. While YW3-56 treatment did not reverse neutrophils increase of peritoneal cavity and peripheral blood caused by LPS, it depressed the activation of neutrophils by inhibiting NET formation, and strikingly decreased TNFα, IL-6, IL-1β, NETs, and cfDNA levels in serum and in the peritoneal cavity, and ultimately alleviated systemic inflammation in LPS-treated mice.

Moreover, inflammation response at the lung induced by LPS endotoxic shock was also attenuated after YW3-56 treatment (Figure 4). Although Liang et al. have demonstrated YW3-56 could reduce NET formation in mouse lungs following LPS exposure [27], they did not elucidate the relationship between NET formation and systemic inflammation. We further studied this pathway from the following aspects. YW3-56 significantly decreased the levels of inflammatory factors TNFα, IL-6, and IL-1β in the serum, BALF, and PLF caused by LPS. Our RNA-seq results further showed that molecule signatures associated with LPS-induced endotoxic shock were largely reversed after YW3-56 treatment in the lung. This indicates that YW3-56 can not only prevent neutrophil netosis by inhibiting the PAD4 activity, but also depress the transcription level of lung inflammatory cytokines, and reduce the systemic inflammatory response in endotoxin shock induced by LPS, thus improving the survival rate of endotoxin shock mice.

Of PAD family members, PAD4 has a nuclear localization signal and localizes mainly to the nucleus to exert its regulatory functions to the chromatin. Our previous studies have found that histone citrullination plays a pivotal role in chromatin decondensation to form NETs [28]. In the PAD4 knockout mouse, we found a lack of NET formation after stimulation with LPS, phorbol myristate acetate (PMA), and calcium ionophore [11]. To date, PAD4 and NETs were found to play a role in etiology of many diseases, including I/R injury of heart, liver, kidney, and intestine, slower wound healing in diabetic mice, and in lung and heart fibrosis of ageing mice [29,30,31,32,33,34].

NETs formed at the site of infection are important for antimicrobial functions. However, an excessive amount of NETs exacerbates local inflammation and tissue damage. As such, the NET formation process must be maintained at a balance of its production and clearance. Various upstream stimulators, including LPS, TNFα, IL-8, and PKC agonists, can activate NET formation [4,35,36]. NETs released to the extracellular space are subject to degradation by DNase to ameliorate their proinflammatory functions [37]. Defects in NET formation and clearance lead to human diseases, such as chronic granulomatous disease (CGD) [38] and atherosclerosis [39], respectively.

LPS binds to TLR4 and activates a cascade of downstream-signaling events that can lead to NET formation and the expression of pro-inflammatory cytokines [40,41]. We found here that PAD inhibitor YW3-56 treatment prevented lethal endotoxin shock induced by LPS administration. YW3-56 inhibits NET formation as well as decreases the levels of inflammatory cytokines detected in the blood, primary, and secondary inflammation sites, including the peritoneal cavity and the lung, respectively. In conclusion, NET inhibition offers a potential treatment strategy for acute and systemic inflammation-associated diseases.

## 4. Materials and Methods

### 4.1. Healthy Blood Donors

Healthy donors were mainly composed of 20–50 year old adults (Table S1). All donors signed and dated an information consent form according to a protocol approved by the Biomedical Scientific Research Ethics Subcommittee of Henan University (HUMSOM-2018-377).

### 4.2. Animals and LPS-Induced Lethal Endotoxic Shock

Female C57BL/6J mice (6–8 weeks old, weighing 17–22 g) were purchased from Beijing Charles River Experimental Animal Technology Co., Ltd. (Beijing, China). All animals were housed in barrier cages under standard laboratory conditions free of specific pathogens and controlled environmental conditions (12/12 h of light/dark cycle, 55 ± 5% humidity, 23 °C) for at least 5 days with food and water ad libitum before the experiment. All experiments were performed in compliance with the animal welfare and research regulations. The animal protocol for this study was approved by the Institutional Animal Care and Use Committee of Henan University.

The mice were randomly divided into three groups (*n* = 6–11/group): (1) Control groups: mice were intravenous injected with dimethyl sulfoxide (DMSO) through the tail vein, followed by intraperitoneal injection with normal saline (NS) after a half-hour. (2) LPS groups: mice were injected with LPS (35 mg/kg, dissolved in NS). (3) LPS + YW3-56 groups: mice were injected with PAD inhibitor YW3-56 (10 mg/kg) which is dissolved in DMSO (0.5 μL/g mouse body weight) at a half-hour before LPS administration. The total number of animals used in the experiment was 219.

In survival observational studies, mice were monitored for 10 consecutive days, then euthanized with chloral hydrate at the endpoint of observation, or whenever they were found moribund. In non-survival studies, animals that received the same treatments as described above were euthanized by chloral hydrate at 24 h after LPS administration. Blood samples were collected from euthanized mouse hearts and kept at room temperature (RT) for 1 h to allow blood clotting. Serum was separated from the clotted blood by centrifugation (3000× *g*, 4 °C) for 20 min, and then stored immediately at −80 °C for further analysis. Lung tissues were harvested and stored at −80 °C for RNA extracting, or fixed in 4% paraformaldehyde in PBS buffer containing 0.1% Triton X-100 for tissue section and immunostaining.

### 4.3. Reagents

Lipopolysaccharide (LPS, L6368), dimethyl sulfoxide (DMSO, MKCP0356), Histopaque-1119 (RNBH0536), 4′,6-diamidino-2-2phenylindole (DAPI, D9542), A23187 (C9275), Nα-Benzoyl-L-arginine ethyl ester hydrochloride (BAEE, B4500), phenylmethanesulfonyl fluoride (PMSF, P7626) were purchased from Sigma-Aldrich (St. Louis, MO, USA). Percoll (17-0891-09) were from the GE Healthcare company (Uppsala, Sweden). Human PAD1 (10784), PAD2 (10785), PAD3 (10786), PAD4 (10500), and mPAD4 (28910) were purchased from Cayman Chemical (Ann Arbor, MI, USA). YW3-56 was synthesized according to previous methods [19] and the purity is >90% estimated by SDS-PAGE. All other inorganic salts reagents were analytically pure and purchased from Solarbio. Roswell Park Memorial Institute (RPMI) 1640 medium (11835030) and fetal bovine serum (FBS) were purchased from Gibco (Grand Island, NY, USA). Rabbit anti-H3cit (2,8,17) (ab5130), Mouse anti-PAD4 (ab128086), Rabbit anti-H3 (ab1791), and anti-rabbit IgG Alexa fluor 568 (ab175471) were purchased from Abcam (Waltham, MA, USA). Mouse anti-GAPDH (60004-1-Ig) were purchased from Proteintech (Wuhan Sanying, Wuhan, Hubei, China). HRP-conjugated anti-rabbit (111-035-003) and HRP-conjugated anti-Mouse (115-035-003) were from Jackson company. APC-labeled anti-mouse CD45 monoclonal antibody (17-0451-82) and FITC-labeled anti-mouse Ly6g monoclonal antibody (11-9668-82) were purchased from Invitrogen (ThermoFisher Scientific, Waltham, MA, USA). APC-labeled anti-human CD16 antibody (#60041AZ) and FITC-labeled anti-human CD66b antibody (#60086FI) were from STEMCELL Technologies.

### 4.4. PAD4 Functional Assay

PAD4 was diluted to 30 nM in assay buffer (20 mM Tris-HCl, 50 mM NaCl, 0.5 mM CaCl_2_, 2 mM DTT, 1 mM PMSF (phenylmethanesulfonyl fluoride), pH 7.6) and added to wells containing various concentrations of compound or DMSO vehicle (0.2% final) in a 96-well transparent plate. Following 60 min preincubation at 37 °C, the reaction was initiated by the addition of substrate BAEE (2.0 mM in final Assay Buffer) and reacted for 90 min at 37 °C. The reaction was quenched by adding 25 μL HClO_4_ solution (5.0 M). After 5 min, 125 μL reagent A (0.2 g diacetyl monoxime and 0.6 g NaCl dissolved in 40 mL H_2_O) and 250 μL reagent B (0.2 g antipyrine, 60 mg FeCl_3_, 10 mL H_2_SO_4_ and 10 mL H_3_PO_4_ dissolved in 20 mL H_2_O) were mixed and the mixture boiled at 100 °C for 30 min, cooled in an ice bath for 5 min. Then absorption at 465 nm was measured by a microplate reader (SynergyTM Neo2, BioTek, Winooski, VT, USA).

### 4.5. Human Blood Neutrophils Isolation

Human neutrophils were obtained from peripheral blood of healthy donors, as aforementioned. Neutrophils were isolated using Histopaque-1119 (Sigma) and Percoll Plus (GE Healthcare, Uppsala, Sweden) gradients, as described [42]; a method that causes minimal activation of neutrophils during isolation. CD66b and CD16 double positive cells by flow cytometry (Figure S5A) and cells with a typical lobulated nuclear morphology by Wright–Giemsa staining (Figure S5B) were identified as neutrophils. The purity of cells was >95%, as shown in both experiment methods. All human blood neutrophils experiments were performed as described above.

### 4.6. Mouse Bone Marrow Neutrophils Isolation

Bone marrow neutrophils were isolated from mice tibias and femurs. Briefly, tibias and femurs were removed and stripped of their muscles. The bone marrow was flushed using PBS buffer, and cell aggregates were disrupted via filtration through a 70-μm cell strainer. Neutrophils were separated by density centrifugation according to the kit instructions (LZS1100, Tianjin, China). Erythrocytes were removed by treatment with erythrocyte Lysing Buffer (0021536, BD Biosciences, Haryana, India).

### 4.7. Immunocytochemistry Staining

Sterile 13 mm round glass cover slips treated with polylysine were placed into 24-well cell culture plates [42]. 2 × 10^5^ isolated human or mice neutrophils were seeded in 500 μL RPMI 1640 medium (containing 2% heat inactivated FBS) or Lock’s solution [43] (10 mM HEPES-HCl 7.3, 150 mM NaCl, 5 mM KCl, 2 mM CaCl_2_, 0.1% glucose) per well and incubated for 2 h with the PAD4 inhibitor YW3-56, or not in CO_2_ incubator at 37 °C. Cells were stimulated with ionomycin A23187 (5 µM) for 2 h and then instantly fixed in 4% paraformaldehyde (PFA) in PBS buffer containing 0.1% Triton X-100. Images of NETs were obtained by staining with rabbit anti-H3Cit (2,8,17) (1:1000, Abcam, ab5103) and anti-rabbit IgG Alexa fluor 568 (1:500, Abcam, ab175471) antibodies, and then captured at excitation 578 nm/emission 603 nm. Nuclear DNA was visualized by DAPI (1:10,000) staining and captured using an inverted fluorescence microscope (Zeiss) at excitation 365 nm/emission 445 nm.

### 4.8. Immunohistochemistry and H & E Staining

Animals were euthanized at 24 h after treatment, and the left and right lung tissues were respectively harvested for immunostaining (*n* = 3) and RNA sequencing (*n* = 3). For staining, the lungs were fixed in 4% PFA for 24 h, and then washed 3 times in PBST (0.1% TritonX-100) for 15 min each time. For section preparation, fixed lungs were immersed successively in 10%, 20%, and 30% sucrose solution overnight, as described [44]; then were embedded by OCT (optimum cutting temperature compound) at −20 °C. Sections of 12-µm-thickness were prepared using a cryostat microtome, and were stained by fluorescence antibodies, as described above, or hematoxylin and eosin (H&E) according to standard protocols [43] and based on the manufacturer’s instructions.

### 4.9. Peritoneal Lavage Fluid (PLF) and Bronchoalveolar Lavage Fluid (BALF) Collection

To analyze the levels of inflammatory factors in PLF and BALF, mice (*n* ≥ 4 per group) were anaesthetized at around 24 h after treatment by intraperitoneally administrating 3 mL cold PBS and gently kneading the abdomen for 3~5 min, and then, PLF was collected. About the same time, BALF was collected by intratracheal administration of 1 mL cold PBS into the lung and pumped back and forth gently 3 times. The PLF and BALF were centrifuged (600× *g*, 10 min, 4 °C), of which the supernatant was collected for cytokines (TNFα, Il-6, IL-1β) and cell-free DNA (cfDNA) detection. The erythrocytes in the precipitation were removed by erythrocyte lysates. The remaining cells were resuspended in PBS (containing 3% BSA) for flow cytometry detection.

### 4.10. Quantitative Real-Time Reverse Transcriptase Polymerase Chain Reaction (RT-PCR)

Total RNA was extracted from lung tissues using TRIzol reagent (15596026, Invitrogen, Carlsbad, CA, USA) at the indicated time points, following LPS administration. cDNA was synthesized from 1 μg RNA by SweScript RT I Enzyme Mix (Servicebio, G3330, Wuhan, China) through incubation for 30 min at 50 °C and 5 s at 85 °C. Each sample was prepared in triplicate in a total reaction volume of 20 μL. cDNA was used after 20-fold dilution in later PCR analyses. Real-time PCR was performed using the SYBR Green qPCR Master Mix (Servicebio, G3321, Wuhan, China). The PCR amplification was performed 2 min at 95 °C for 1 cycle, 5 s at 94 °C, 5 sec at 55 °C and 5 s at 72 °C for 40 cycles, and 30 s at 72 °C for 1 cycle. All reactions were performed using a Quant Studio 5 Real-Time PCR system. Primer sequences were listed in Table S2. Beta actin was used as an internal control gene, and relative gene expression was calculated using the comparative threshold cycle (*Ct*) method by the following equation:

$$2^{-\Delta \Delta Ct}=2^{-(\Delta Ct1-\Delta Ct2)}=2^{-[(T-C)-(U-C)]}$$

### 4.11. Western Blot Analysis

Western blot analyses were performed essentially, as described previously [43]. 3 × 10^6^ isolated human or mice neutrophils that were treated with A23187 only or both YW3-56 and A23187, as described above, were homogenized in IP buffer (20 mM Tris-HCl, [pH 8.0], 150 mM NaCl, 10 mM EDTA, 0.2% Triton X-100, 0.2% NP-40, 1 mM PMSF, 1.5 μg/mL aprotinin, 1 μg/mL Leupeptin, 1 μM Pepstain). The concentration of total proteins was quantified by the Bradford method. Then, 20 μg total proteins were separated in 13% SDS-PAGE gels and electro transferred to nitrocellulose or PVDF membranes and blocked for 30 min with 5% fat free dry milk in TBST (1 × TBS with 0.1% Tween 20) before the primary antibodies were applied. Antibodies used in Western blot were Mouse anti-PAD4 (1:5000, Abcam, ab128086), Rabbit anti-H3Cit3 (1:10,000, Abcam, ab5103), Rabbit anti-H3 (1:10,000, Abcam, ab1791), Mouse anti-GAPDH (1:6000, Proteintech, 60004-1-Ig), HRP-conjugated anti-rabbit (1:10,000, Jackson, 111-035-003), HRP-conjugated anti-Mouse (1:10,000, Jackson, 115-035-003). After overnight incubation at 4 °C with primary antibodies and washing, corresponding HRP-conjugated secondary antibodies were incubated for 2 h at room temperature, followed by washing. The HRP signals were detected by enhanced chemiluminescence reagents and imaged by a super-sensitive multifunctional imaging instrument (Amersham Imager 680UV, GE).

### 4.12. NETs, cfDNA and Cytokines Analysis in Serum, PLF and BALF

Levels of NETs in serum were analyzed by Elisa. Elisa plates were coated with anti-H3Cit3 antibody (150 ng/well, ab5103) overnight at 4 °C. Then serum samples were incubated for about 2 h and rinsed 3 times for 3~5 min each time. POD-conjugated anti-DNA antibodies (Cell Death Detection ELISAPLUS, 11774425001, Roche, Hong Kong, China) were added to allow the antibodies to bind to the DNA-H3Cit3 complex, and then substrates were added to show color. The reaction was terminated with H_2_SO_4_, and OD 405 was detected by a microplate reader (SynergyTM Neo2, BioTek, USA).

The amount of cfDNA in serum, PLF, and BALF was measured by the INVITROGEN Qubit3.0 Fluorometer (Thermofisher, Waltham, MA, USA) using the Qubit™ dsDNA HS Assay Kit (Invitrogen, Q32854) according to the manufacturer’s instructions.

The levels of TNFα, IL-6, IL-1β, and HMGB1 in serum, PLF, and BALF were quantified using the ELISA kit from Boster Biological Technology Company (Wuhan, China) and Elabscience Biotechnology Company (Wuhan, China) according to the manufacturer’s instructions.

### 4.13. Flow Cytometry Detection

To analyze the quantity and ratio of neutrophils, isolated cell pellets in PLF or BALF were washed with cold PBS and resuspended in 100 μL of flow buffer (0.04% BSA in PBS). Then, cells were incubated with APC-labeled anti-human CD16 antibodies (STEMCELLTM, #60041AZ), FITC-labeled anti-human CD66b antibodies (STEMCELLTM, #60086FI), APC-labeled anti-mouse CD45 monoclonal antibodies (Invitrogen, 17-0451-82), or FITC-labeled anti-mouse Ly6g antibodies (Abcam, ab25024) at 4 °C for 30 min to allow binding. Upon removal of unbound antibodies by washing of the cells with PBS twice, cells were resuspended in flow buffer and placed in 4 °C and blocked from light prior to measurement. All flow-cytometry-based assays were performed on a Beckman CytoFLEX. Data were analyzed using CytoExpert software.

To detect the proportion and apoptosis of neutrophils in peripheral blood of mice, blood samples were collected from anesthetized mouse hearts. Red blood cells were lysed at 4 °C for 10 min. Leukocytes from the peripheral blood were collected after centrifugation. Cell pellets were washed with cold PBS and resuspended in flow buffer. Then, cells were incubated with APC-labeled anti-mouse CD45 monoclonal antibodies and FITC-labeled anti-mouse Ly6g antibodies at 4 °C for 30 min to allow binding. The cell size and granularity of neutrophils were determined based on the CD45 and Ly6G double positive cells. Apoptosis of this cell population was analyzed using Annexin V-FITC Apoptosis Detection Kit (C1062, Beyotime Biotechnology).

### 4.14. RNA Sequencing Analyses

About twenty-four hours after the administration, three mice were randomly selected from each group, and one side of their lung lobe was taken for RNA-seq analysis. The total RNA of each sample was extracted using TRIzol Reagent/RNeasy Mini Kit (Qiagen). Total RNA was quantified and qualified by Agilent 2200 Bioanalyzer (Agilent Technologies, Palo Alto, CA, USA). 1 μg total RNA was used for library preparation. Next generation sequencing library preparations were constructed according to the manufacturer’s instructions. Then, libraries with different indices were multiplexed and loaded on an Illumina Novaseq instrument according to manufacturer’s instructions (Illumina, San Diego, CA, USA). Sequencing was carried out using a 2 × 150 paired-end (PE) configuration. Image analysis and base calling were conducted by the NovaSeq Control Software (NCS) + OLB + GAPipeline-1.6 (Illumina) on the NovaSeq instrument. The sequences were processed by GENEWIZ (Suzhou, China).

Reads were filtered for quality using the fastqc (version 0.11.9) and trim_galore (version 0.6.7) software and aligned to the ENSEMBL reference genome Mus_musculus GRCm39 using the Hisat2 software (version 2.2.1). Gene expression quantities were analyzed by the featureCounts software (version 2.0.1).

Differentially expressed genes (DEGs) were analyzed using R software (4.0.5) with the DESeq2 Bioconductor package. DEG was identified as a gene with a fold change over 2 and *p*-value less than 0.05. GO enrichment and KEGG pathways annotation were analyzed by using the clusterProfiler package.

### 4.15. Statistical Analysis

Distribution of the continuous variables was assessed using the Kolmogorov–Smirnov test. Depending on the normality, all continuous data were expressed as the mean ± standard error of mean (SEM) and statistical analyses were performed using the GraphPad Prism 8.0. (GraphPad Software Inc., La Jolla, CA, USA). Unpaired t test was used to analyze significant differences of the data between the groups. Kaplan–Meier curves and log-rank tests were performed to analyze the survival curve. Pearson’s correlation was used to estimate the association between NETs and cfDNA in serum. All the experiments were conducted independently with at least four replicates. *p* < 0.05 were considered statistically significant. The post hoc power analysis was performed using the G*Power software (Heinrich Heine University, Düsseldorf, Germany).

## Data Availability

All data are available in the main text and the supplementary materials. The RNA-sequencing data presented in this study has been deposited in GEO under accession No. GSE203340.

## Supplementary Materials

The following supporting information can be downloaded at: https://www.mdpi.com/article/10.3390/ijms232113264/s1.
