# Supplementary material for: Inhibition of Netosis with PAD Inhibitor Attenuates Endotoxin Shock Induced Systemic Inflammation
Source: Int J Mol Sci. 2022 Oct 31;23(21):13264. doi: 10.3390/ijms232113264 (PMC9655899; doi:10.3390/ijms232113264)
Supplement: Supplementary file 1 [file ijms-23-13264-s001.zip › ijms-1951440-supplementary.pdf]

Supplementary Figures and Figure Lengends

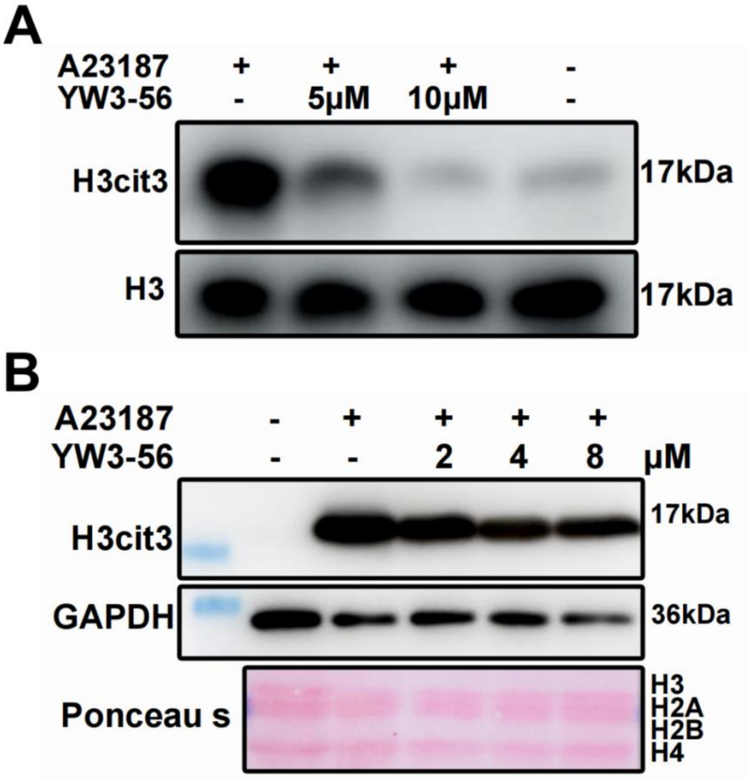

Figure S1. PAD inhibitor YW3-56 inhibits histone citrullination and NET formation of human and mouse neutrophils by Western blot. (A) Upon calcium ionophore A23187 treatment, human peripheral blood neutrophils increased histone citrullination. Histone citrullination were decreased upon YW3-56 treatment. (B) Mouse bone marrow neutrophils showed a dramatic increase in histone citrullination after calcium ionophore treatment. YW3-56 treatment blocked the increase in histone citrullination.

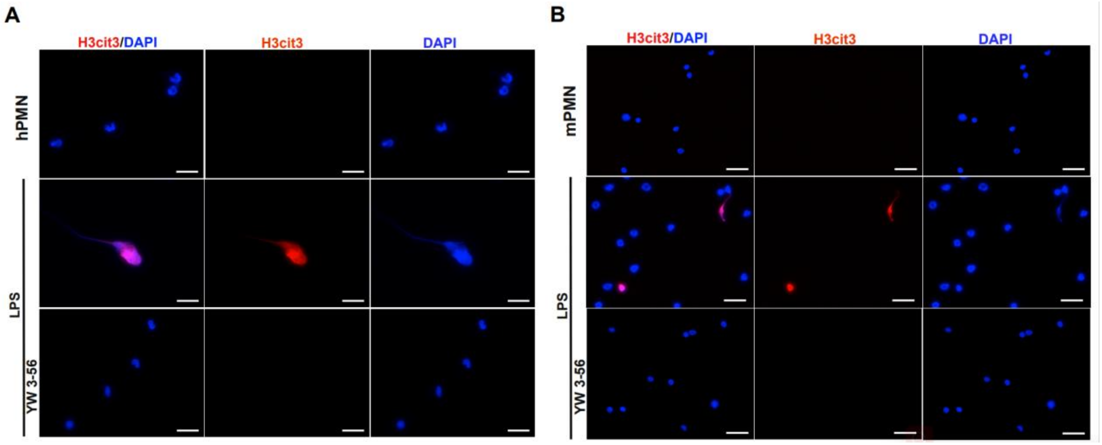

Figure S2. PAD inhibitor YW3-56 inhibits histone citrullination and NET formation of human and mouse neutrophils induced by LPS treatment. (A) Upon LPS treatment, human peripheral blood neutrophils increased histone citrullination and NET formation (middle, panels) detected with immunostaining with a histone H3Cit (Cit2, 8, 17) antibody. DNA dye DAPI staining in blue. NET formation and histone citrullination were decreased upon YW3-56 treatment. (B) Mouse bone marrow neutrophils showed a dramatic increase in histone citrullination after LPS treatment. YW3-56 treatment blocked the increase in histone citrullination. Scale bars represent 20 μm. hPMN, human polymorphonuclear cell; mPMN, mouse polymorphonuclear cell; PAD, peptidylarginine deiminase; NET, neutrophil extracellular trap; LPS, lipopolysaccharides.

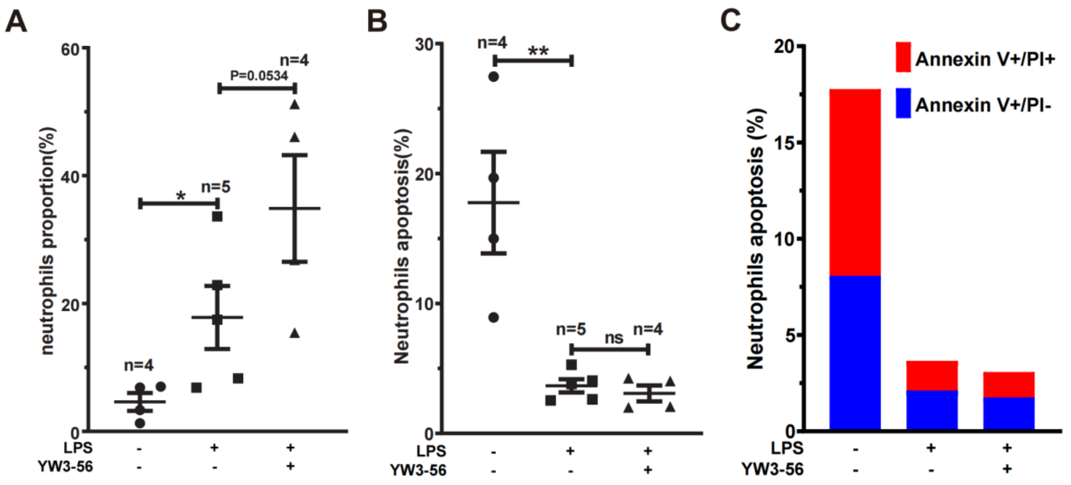

Figure S3. The proportion and apoptosis of neutrophils in blood at different treatment groups. (A) The proportion of neutrophils in leukocytes significantly increased after LPS treatment, and YW3-56 further enhanced it (n = 4, 5, 4), which was consistent with that the proportion of neutrophils in peritoneal lavage fluid increased after LPS treatment and YW3-56 further enhanced it (Figure 3F). (B) The apoptosis of neutrophils significantly decreased in peripheral blood of mice after LPS treatment, and YW3-56 didn't reverse this decrease (n = 4, 5, 4). (C) Neutrophils apoptosis was identified by Annexin V and PI. All data in figures were presented as mean ± SEM. *$P < 0.05$; **$P < 0.01$; ns, no significance. LPS, lipopolysaccharides.

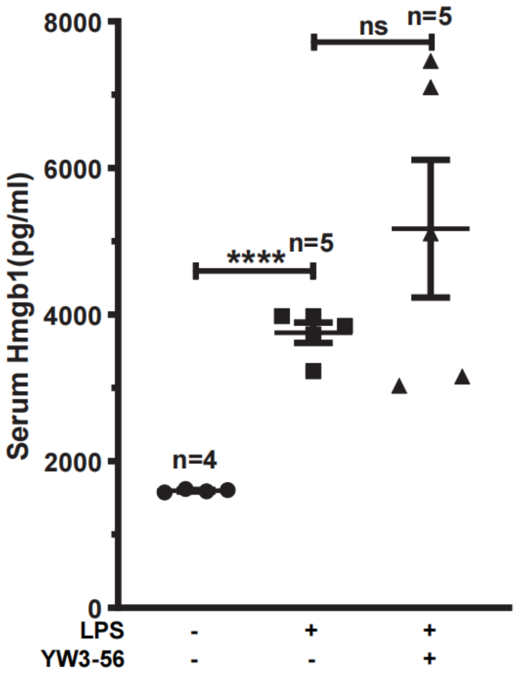

Figure S4. Serum HMGB1 levels were much increased after LPS treatment, while PAD inhibitor YW3-56 treatment didn't decrease HMGB1 levels (n = 4, 5, 5). All data in figures were presented as mean ± SEM. ****$P < 0.0001$. HMGB1, high mobility group protein B1.

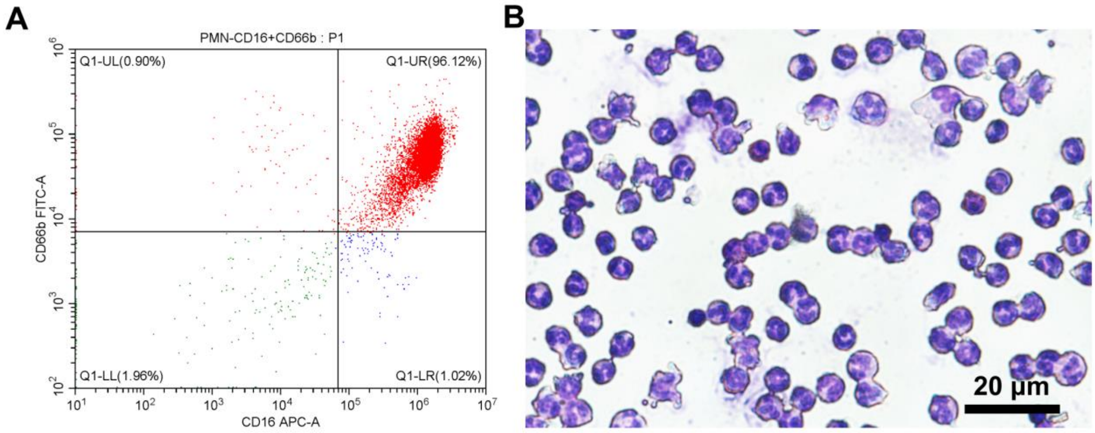

Figure S5. The purity of isolated human peripheral blood neutrophils. (A) The purity of neutrophils labeled as CD66b$^+$ and CD16$^+$ double positive in isolated peripheral blood polymorphonuclear cells had greater than 95%. (B) Isolated peripheral blood polymorphonuclear cells had a typical lobulated nuclear morphology by Wright-Giemsa staining. Scale bars represent 20 μm.

Table S1: Details of the healthy donors

Gender	Age	Number
women	20-30	27
women	31-40	3
women	40-50	1
men	20-30	21
men	31-40	4
men	40-50	2

Table S2: primer index

Genes	Primer Direction	Primer Sequences
Actin	Forward	GTTGGAGCAAACATCCCCCA
Actin	Reverse	ACGCGACCATCCTCCTCTTA
Tnfα	Forward	CCTCTCATCAGTTCTATGGC
Tnfα	Reverse	CACCACTAGTTGGTTGTCTT
Csf3	Forward	ATGGCTCAACTTTCTGCCCAG
Csf3	Reverse	GGGGAAATACCCGATAGAGCC
Il1β	Forward	CTGTGACTCATGGGATGATGATG
Il1β	Reverse	CGGAGCCTGTAGTGCAGTTG
Il6	Forward	CTGCAAGAGACTTCCATCCAG
Il6	Reverse	AGTGGTATAGACAGGTCTGTTGG
Il10	Forward	CTTACTGACTGGCATGAGGATCA
Il10	Reverse	GCAGCTCTAGGAGCATGTGG
Ccl2	Forward	TTAAAAACCTGGATCGGAACCAA
Ccl2	Reverse	GCATTAGCTTCAGATTTACGGGT
Cxcl9	Forward	TCCTTTTGGGCATCATCTTCC
Cxcl9	Reverse	TTTGTAGTGGATCGTGCCTCG
Cxcl10	Forward	CCAAGTGCTGCCGTCATTTTC
Cxcl10	Reverse	GGCTCGCAGGGATGATTTCAA
Nfκb	Forward	AGAGGGGATTTCGATTCCGC
Nfκb	Reverse	CCTGTGGGTAGGATTTCTTGTTC
Nfκbia	Forward	TGAAGGACGAGGAGTACGAGC
Nfκbia	Reverse	TTCGTGGATGATTGCCAAGTG
Stat3	Forward	CAATACCATTGACCTGCCGAT
Stat3	Reverse	GAGCGACTCAAACTGCCCT
Padi4	Forward	AAGGGCTACACAACCTTCGG
Padi4	Reverse	GCTGCTTTCACCTGTAGGGT
Ly6g	Forward	GACTTCCTGCAACACAACTACC
Ly6g	Reverse	ACAGCATTACCAGTGATCTCAGT
Mpo	Forward	GACATGCCCACCGAATGACAA
Mpo	Reverse	CAGGCAACCAGCGTACAAAG
Elane	Forward	CAGGAACTTCGTCATGTCAGC
Elane	Reverse	AGCAGTTGTGATGGGTCAAAG
